# Phosphorylation and Circadian Molecular Timing

**DOI:** 10.3389/fphys.2020.612510

**Published:** 2020-11-26

**Authors:** Andrea Brenna, Urs Albrecht

**Affiliations:** ^1^Department of Medicine, University of Fribourg, Fribourg, Switzerland; ^2^Department of Biology, University of Fribourg, Fribourg, Switzerland

**Keywords:** phosphorylation, circadian, kinase, clock, metabolism

## Abstract

Endogenous circadian rhythms are biological processes generated by an internal body clock. They are self-sustaining, and they govern biochemical and physiological processes. However, circadian rhythms are influenced by many external stimuli to reprogram the phase in response to environmental change. Through their adaptability to environmental changes, they synchronize physiological responses to environmental challenges that occur within a sidereal day. The precision of this circadian system is assured by many post-translational modifications (PTMs) that occur on the protein components of the circadian clock mechanism. The most ancient example of circadian rhythmicity driven by phosphorylation of clock proteins was observed in cyanobacteria. The influence of phosphorylation on the circadian system is observed through different kingdoms, from plants to humans. Here, we discuss how phosphorylation modulates the mammalian circadian clock, and we give a detailed overview of the most critical discoveries in the field.

## Introduction

**Figure 1:**
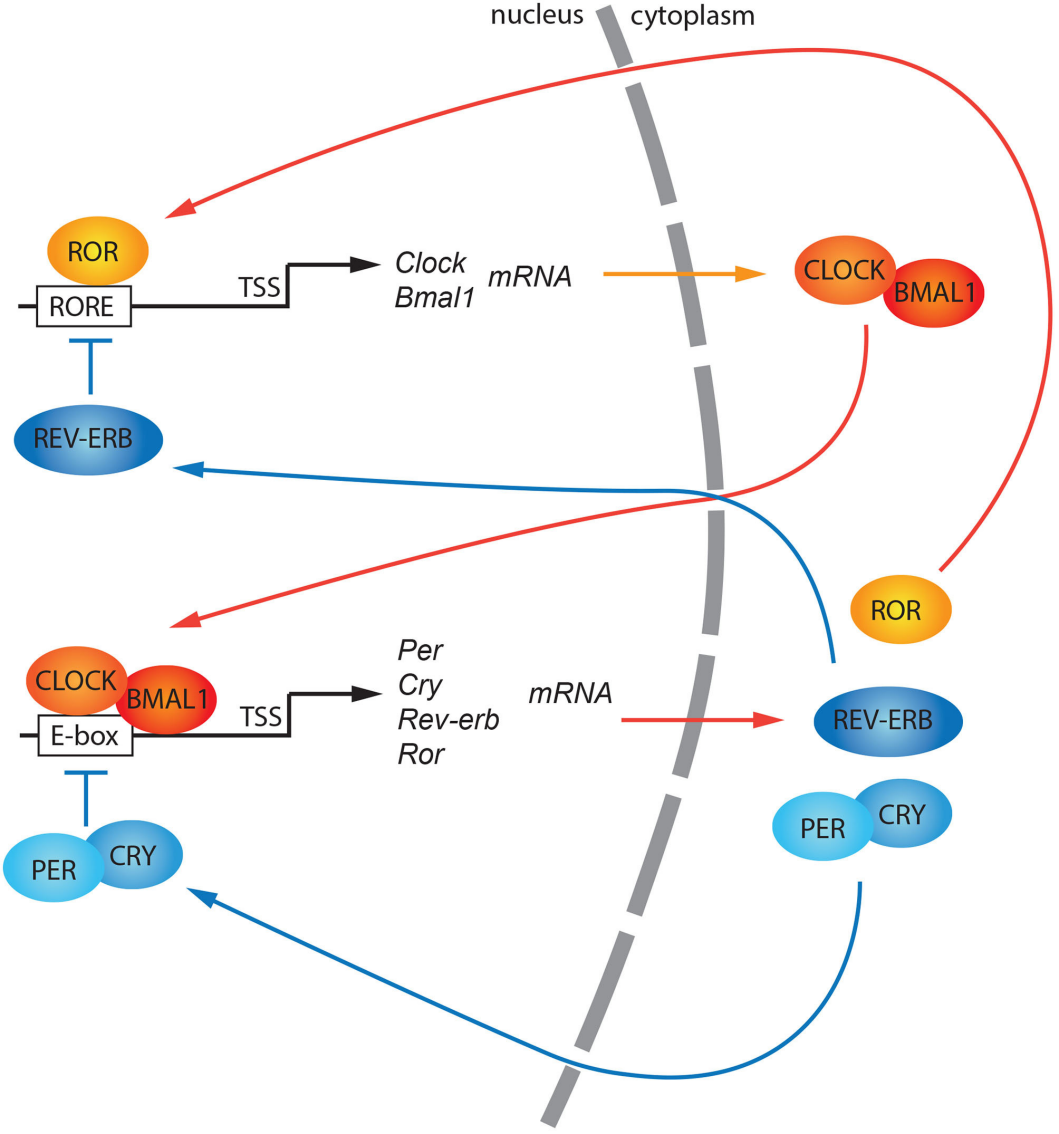
A general model of the canonical circadian molecular feedback loop. CLOCK and BMAL1 promote the expression of *clock-controlled genes* (*ccgs*), and genes encoding PERs and CRYs. PER (1, 2, 3) and CRY (1, 2) are progressively accumulated in the cytoplasm and subsequently shuttled into the nucleus as hetero/homodimers. Here, they repress the CLOCK: BMAL1 transactivation. *Rev-erb* and *Ror* gene expression is also regulated by CLOCK: BMAL1 transactivation. Both their protein products, REV-ERB, and ROR, compete for the *ROR responsive elements* (*ROREs*) within the *Bmal1* promoter driving its circadian gene expression. TSS, Transcriptional Start Site.

Circadian rhythms consist of adaptive physiological responses, which allow organisms to anticipate changes in light-dark cycle that occurs within 24-h. Exogenous stimuli (also referred to as zeitgebers or time givers), such as light, temperature, and nutrients, can entrain organisms to the 24-h cycle (Schibler and Sassone-Corsi, 2002). At the evolutionary level, regular changes in light and temperature around the day have favored the formation of genetic circuits, called internal circadian clocks. These clocks can keep track of daily changes and prepare the organism to adapt to them (Panda et al., 2002b). Biological clocks are observed from prokaryotes to mammals, suggesting that these mechanisms are highly conserved across the kingdoms (Bell-Pedersen et al., 2005). At the molecular level, the internal circadian clock can be described as a transcriptional-translational feedback loop (TTFL) (Dunlap et al., 1999). In mammals, the heterodimer of two basic helix-loop-helix/PAS domain-containing transcription factors, CLOCK: BMAL1 (or NPAS2: BMAL1), orchestrate this regulatory feedback loop by binding the *E-boxes* present on promoters of other clock genes such as *Per* (*Per1, Per2, Per3*) and *Cry* (*Cry1, Cry2*) genes. *Per* and *Cry* mRNAs are translated into proteins in the cytoplasm, and subsequently, they go back into the nucleus as PER homodimers (Kucera et al., 2012) and PER:CRY heterodimers or CRY monomers (Chiou et al., 2016). In the nucleus, they suppress the CLOCK: BMAL1 transcriptional activity regulating their accumulation (Buhr and Takahashi, 2013) (Figure 1). Also, the nuclear receptors belonging to REV-ERB and ROR families play a role in stabilizing the robustness of the feedback loop controlling the oscillation of *BMAL1* gene expression (Preitner et al., 2002; Akashi and Takumi, 2005; Takeda et al., 2012; Ikeda et al., 2019) (Figure 1).

**Figure 2:**
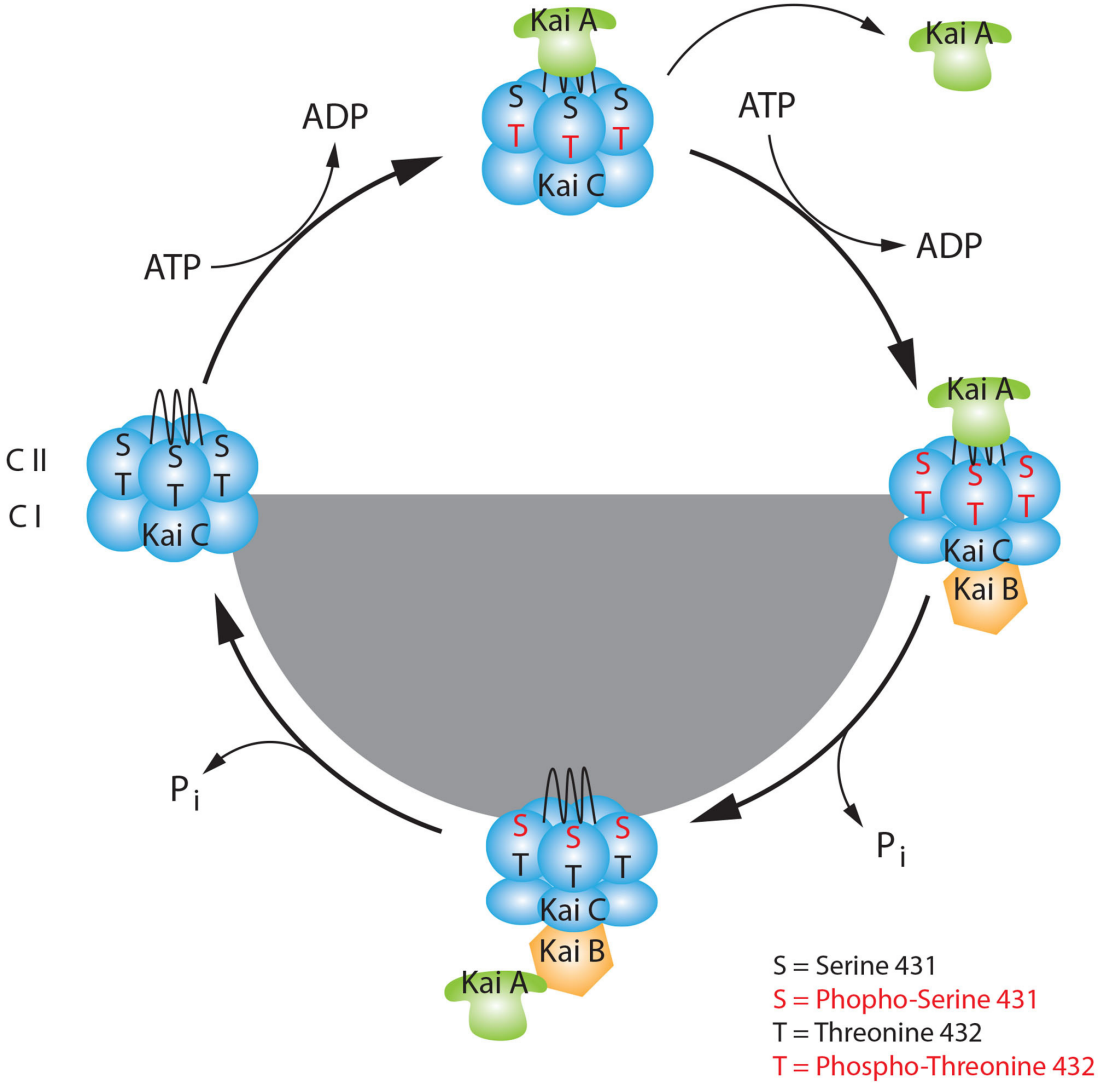
The basic model of the KaiABC circadian oscillator. KaiC consists of two double donut hexamers (CI, CII) one fused one on the top of the other. During the day, the KaiC upper hexamer can interact with KaiA through its tentacular A-loop and it can be phosphorylated at Threonine 432 (Thr-432), in presence of ATP. The first set of phosphorylation at Thr-432 triggers the subsequent phosphorylation at Serine 431 (Ser-431), which favors the binding to KaiB at dusk. This event causes a change of conformation in the KaiC quaternary structure. Here, during the night phase, KaiB can sequestrate KaiA, and KaiC starts the auto dephosphorylation process. During the late-night, when KaiC is hypophosphorylated at sites Thr-432 and Ser-431 it releases KaiA and KaiB. This event leads to a new change of conformation of KaiC which makes it ready for a new cycle.

In mammals, the circadian rhythmicity is modulated by the suprachiasmatic nucleus (SCN) of the hypothalamus. The SCN coordinates, comparable to an orchestra's conductor, the “tempo” of the clocks placed all over the body (Reppert and Weaver, 2002). Thereby, the cellular feedback loop described above is responsible for the oscillation of about 10–20% of all genes expressed (Panda et al., 2002a; Storch et al., 2002). Nevertheless, 20% of oscillating proteins do not show signs of rhythmicity at the mRNA level. This evidence suggests that the transcriptional feedback loop is not the only mechanism involved in the generation of rhythmicity in living organisms (Robles et al., 2014). In addition to transcriptional regulation, many different levels of regulation can be taken into consideration, such as chromatin remodeling, post-transcriptional, translational, and post-translational modifications (Aguilar-Arnal and Sassone-Corsi, 2013; Kojima and Green, 2015; Michael et al., 2015; Hirano et al., 2016). Of particular interest in this review are post-translational modifications (PTMs), which are covalent modifications on proteins that confer specific new features to them. These modifications influence cellular localization, protein-protein interactions, and protein stability (Mehra et al., 2009). The most ancient example of circadian rhythmicity is present in *cyanobacteria*. In this organism, in contrast to the mammalian TTFL, the circadian clock is generated and driven by a post-translational oscillator (PTO) (Iwasaki et al., 2002; Nakajima et al., 2005; Chang et al., 2015; Tseng et al., 2017). The PTO consists of three different proteins, KaiA, KaiB, and KaiC. KaiC is provided with autokinase and autophosphatase activity. During the light phase, KaiA can bind and stabilize KaiC, promoting KaiC autophosphorylation at residues Ser-431 and Thr-432. This process is counteracted by KaiB, which sequesters KaiA at night, promoting KaiC autodephosphorylation. The counterbalanced activity of both KaiA and KaiB on KaiC is responsible for the diurnal oscillation of the phosphorylation state (Figure 2). Since mammalian phosphorylation of clock proteins have also been observed, it appears that phosphorylation is a highly conserved PTM across kingdoms involved in the generation of circadian rhythmicity (Baker et al., 2009; Reischl and Kramer, 2011). Phosphorylation is a reversible PTM that occurs on serine (90%), threonine (10%), and tyrosine (0.05%) residues of amino acids. This dynamic change can produce a fast and precise alteration of protein characteristics, which in turn affects many biological processes (Seet et al., 2006). Here, we will give an overview of some of the important discoveries made in the last decades about how phosphorylation can regulate the circadian clock in mammals and how this is coupled to physiological responses. Finally, we will discuss the involvement of circadian phosphorylation in diseases.

## Light: Dark Driven Phosphorylation of the Clock Machinery

### The Positive Loop

The heterodimer CLOCK: BMAL1 is the core complex of the positive loop that regulates the circadian transcription in mammals. It displays a maximal promoter occupancy of clock-controlled genes between ZT5 and ZT8 (Ripperger and Schibler, 2006). However, levels of CLOCK and BMAL1 total proteins do not oscillate dramatically. Thus, protein oscillation is not sufficient to justify such a tight temporal profile of nuclear accumulation and promoter occupancy. This evidence implies that PTMs are required to dictate the precise temporal/spatial organization of the circadian positive core complex. Some observations indicated that the phosphorylation of the CLOCK protein is relevant. First, the oscillatory profile of CLOCK phosphorylation was peaking at ZT18, and this modification was able to trigger its degradation (Yoshitane and Fukada, 2009). Second, Clock Δ19 mutant mice, which contain a deletion of exon 19, display a prolonged period compared to wild type controls (King et al., 1997). Third, the phosphorylation level of CLOCK Δ19 was lower than the wild type, and the protein was more stable.

Additionally, Clock Δ19 showed a weaker transactivation activity, without affecting its heterodimerization with BMAL1 (King et al., 1997). These observations led scientists to study and map putative CLOCK phosphorylation sites. Two interesting phosphorylation sites were mapped on Serine-38/S42 (Ser-38/42) located in the basic Helix-Loop-Helix (bHLH) domain (Yoshitane et al., 2009). The results indicated that these specific phosphorylations were a marker for a two-step process. First, CLOCK transactivation activity is inhibited when the protein is phosphorylated at those sites. Second, double phosphorylation at Ser-38/42 is a signal for protein translocation to the cytoplasm.

On the other hand, recent studies on the circadian liver phosphoproteome found CLOCK phosphorylation at sites Ser-446 and Ser-440/441, which increase transcriptional activity (Robles et al., 2017). Additionally, there is evidence supporting that the cytoplasmic-nuclear distribution of CLOCK is regulated by Cyclin-Dependent Kinase 5 (CDK5) (Kwak et al., 2013).

**Figure 3:**
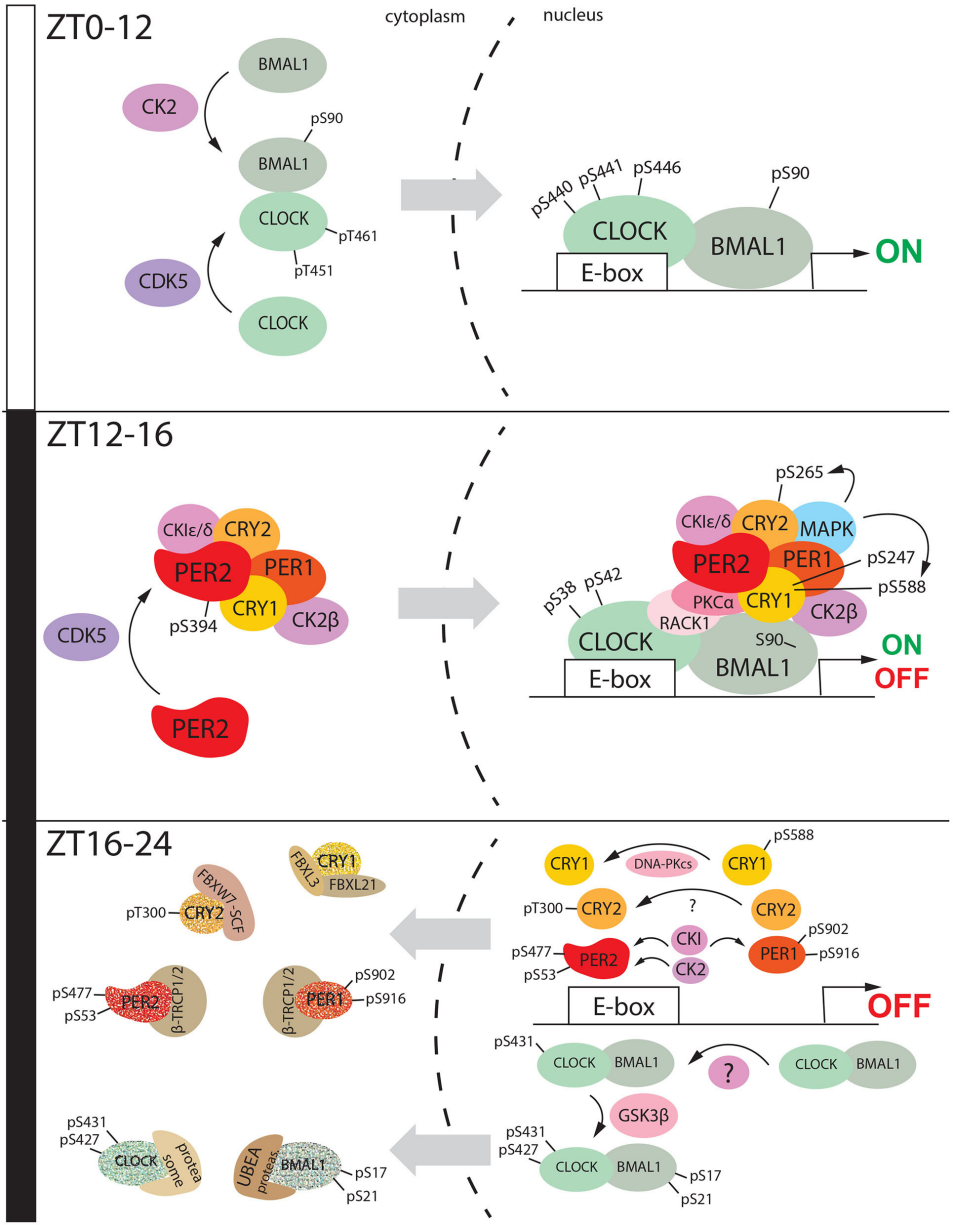
The diurnal nucleus/cytoplasm shuttling of clock factors is mediated by phosphorylation. During the day (ZT0-ZT12), Clock is phosphorylated by CDK5 at specific sites Threonine 451 and 461, while BMAL1 is phosphorylated by CK2 at Serine 90. Somehow these events can promote heterodimerization and shuttle from the cytoplasm to the nucleus. Here, CLOCK is phosphorylated at Serine 440-441-446, and this post-translational modification promotes the transactivation mediated by CLOCK: BMAL1. During the early night (ZT12-16), CDK5 mediated phosphorylation of PER2 at Serine 394. This event promotes heterodimerization with CRY1 and probably, the assembling of the cytoplasmic macromolecular negative complex consisting also of PER1, CRY1, CK2β, and CKI ε/δ. This macromolecular complex goes into the nucleus and it complexes with the CLOCK: BMAL1 heterodimer. This event is promoted by phosphorylation of CRY1 at Serine 247 and 588, CRY2 at Serine 265. CLOCK is phosphorylated at Serine 38 and 42 which leads to the transactivation shut down. During the late-night (ZT16,-20), the nuclear macromolecular complex is disassembled. CLOCK undergoes subsequent phosphorylation at Serine 431 and 427, the second one mediated by GSK3β, which also phosphorylates BMAL1 at Serine 17 and 21. This even triggers CLOCK: BMAL1 shuttling to the cytoplasm followed by proteasomal degradation. CKI can phosphorylate both PER2 and PER1, respectively at Serine 477 (PER2) and Serine 902/916 (PER1). PER2 is additionally phosphorylated by CK2 at serine 53. All these events promote PER1/PER2 degradation in the cytoplasm mediated by β-TRCP1/2. CRY1 is translocated into the nucleus after being dephosphorylated at Serine 588. Here it is degraded by the proteasomal complex formed by FBXL3 and FBXL21. On the other hand, CRY2 is phosphorylated at Threonine 300, and this modification drives its degradation mediated by FBXW7-SCF. All these events between ZT16 and ZT24 close the 24 h cycle.

These observations suggest a three-step regulation of CLOCK by phosphorylation. First, CDK5-mediated phosphorylation at Threonine 451/461 (Thr-451/461) promotes CLOCK nuclear localization. Here, modifications at Ser-446 and Ser-440/441 increase CLOCK transactivation activity (Figure 3, ZT0-12), and subsequently, modifications at Ser-38/42 shut down the gene expression (Figure 3, ZT12-16). However, none of these phosphosites is associated with known kinases. Still, CLOCK-interacting protein circadian (CIPC), which is an additional negative-feedback regulator of the circadian clock, modulates the phosphorylation at Ser-38/42 (Yoshitane and Fukada, 2009). While the phosphorylation at Ser-38/42 is associated with loss of transactivation and cytoplasmic retention of CLOCK, it does not explain the subsequent degradation of the protein. A further investigation led to the identification of specific clusters of amino acids (aa 425-461) involved in the degradation of CLOCK. Phosphorylation at Ser-427 by Glycogen Synthase Kinase 3 Beta (GSK3β), which requires a priming kinase, is involved in the regulation of CLOCK degradation (Spengler et al., 2009). It has been shown that the Ser-431 is the priming site and, although the kinase involved in this particular modification is unknown, results show that the specific phosphorylation is BMAL1-dependent. BMAL1-dependent GSK3β phosphorylation of CLOCK at Ser-427 leads to protein degradation via the proteasome (Figure 3, ZT16-24). Thus, the CLOCK bound to E-boxes can be removed for the next round of transcriptional activation, closing the transcriptional feedback loop. The promoter clearance is a necessary step to keep the circadian profile of gene expression finely modulated and “on time.” Altogether, these pieces of evidence highlight the importance of CLOCK turnover and localization in the regulation of the circadian clock.

When the first circadian genes were discovered, high efforts were made to define whether enzymes, called periodically fluctuating kinases (PFKs), could regulate circadian proteins (Tamaru et al., 1999). Among them, p45^PFK^, a serine/threonine-protein kinase later identified as Casein Kinase 2α catalytic subunit (CK2α), was able to phosphorylate BMAL1 at Serine 90 (Ser-90), which displayed circadian oscillation, promoting cytoplasm/nuclear translocation (Tamaru et al., 2009). This phosphorylation is a prerequisite for the CLOCK: BMAL1 heterodimerization and transactivation (Figure 3, ZT0-12). Loss of phosphorylation at Ser-90 showed arrhythmic activity in mice fibroblasts. Additionally, suppression of this specific phosphorylation in the SCN led to dampening circadian gene expression, suggesting the involvement of pSer-90 in the BMAL1-dependent transcription (Tamaru et al., 2015). These observations demonstrated that the phosphorylation of BMAL1 at Ser-90 was crucial for the central and peripheral clock. Results obtained from different groups suggest that pSer-90 promotes acetylation of BMAL1 at Lysine-537, which is a prerequisite for BMAL1 to recruit CRY1 (Tamaru et al., 2009).

This specific modification at Ser-90 is suppressed by CRY1-mediated periodic binding of BMAL1 with CK2β. Indeed, the heterodimer BMAL1: CRY1 promotes the association between BMAL1 and CK2β, which leads to a decrease of phosphorylation at Ser-90. This event is followed by inhibition of transcriptional activation of clock-controlled genes. These combined processes are responsible for the circadian oscillation of this specific modification.

When pSer-90 goes down, and the repressive phase starts, BMAL1 complexes with RACK1 and Ca^2+^-sensitive protein kinase C subunit α (PKCα) that leads to the inhibition of the transactivation activity (Robles et al., 2010) (Figure 3, ZT12-16).

Subsequently, GSK3β phosphorylates BMAL1 at Ser-17/ Thr-21 and priming it for ubiquitination (Sahar et al., 2010). Protein degradation follows the ubiquitination of BMAL1 through the HECT-type E3 ligase (UBE3A) (Gossan et al., 2014) (Figure 3, ZT16-20).

### The Negative Loop

The so-called “nuclear PER complex,” with a mass of circa 1.9 Mda is a complex based on the physical interaction between, at least, PER1-3, CRY 1-2, Casein Kinase I δ/ε (CKI δ/ε), which functions as repressor machinery of the circadian clock (Aryal et al., 2017). It is recruited onto the heterodimer CLOCK: BMAL1. It is responsible for turning off transcription and dissociation of CLOCK: BMAL1 from the chromatin, thereby closing the transcriptional-translational loop. A critical aspect of the formation of the repressive complex is the profound time delay of several hours between protein production and nuclear accumulation (Vanselow and Kramer, 2007). Additionally, PER proteins show a large amplitude in the oscillation of phosphorylation levels in both the central and peripheral clock (Lee et al., 2001). This evidence highlights the need to better understand the role of phosphorylation in the generation of circadian rhythmicity.

PER2 nuclear entry is favored by heterodimerization with CRY1 (Miyazaki et al., 2001; Chaves et al., 2006). For many years it was unknown whether phosphorylation could regulate this step of the circadian clock. Recent evidence suggests that CDK5 is likely to be involved in this process (Brenna et al., 2019). This kinase can phosphorylate PER2 in the SCN at Serine 394 (Ser-394) in a circadian fashion. The peak of this specific modification reaches the maximum at ZT12, at the transition between the light and dark phase. Additional experiments demonstrated that PER2 pSer-394 is more prone to bind CRY1, and unphosphorylated PER2 is confined to the cytoplasm and eventually degraded. The PER2: CRY1 cytoplasmic complex also comprises PER1 and CKI, which is also a candidate to be the target of CDK5 at the specific site Thr-347, as evidenced *in vitro* (Eng et al., 2017). Thus, CDK5 might be the kinase priming the formation of the large cytoplasmic complex, described by Aryal et al. (2017) (Figure 3, ZT0-12). This finding explains the genesis of the negative loop in the mammalian circadian clock. Finally, it has been proposed that also GSK3β might influence PER2 nuclear accumulation independently by the interaction with CRY1 (Iitaka et al., 2005).

PER2 nuclear translocation oscillates during the day. In the SCN, the total PER2 protein starts to rise at ZT 8 (Brenna et al., 2019) and peaks at ZT 16 (Nam et al., 2014), where it interacts with CKI δ/ε (Figure 3 12-16). It has been reported that CKIε targets PER2 to the proteasomal degradation (26S proteasome), via interaction with a member of the Skp-Cullin-F box (SCF) E3 ubiquitin ligase complex containing Beta-transducin repeats-containing proteins 1-2 [β-TrCP1-2 (Reischl et al., 2007; Ohsaki et al., 2008)]. These specific E3 ligases need priming phosphorylation to bind the targets called phosphodegrons. CKI phosphorylates the Serine-477 (Ser-477) of PER2, priming the binding sites recognized by β-TrCP (Eide et al., 2005). Finally, PER2 is also phosphorylated by CKII2 at Serine-53 (Tsuchiya et al., 2009). However, the importance of this site is still unknown, although it has been proposed that CKII might support CKI in the regulation of the PER2 degradation pathway.

Many pieces of evidence suggested that CK1ε can promote the turnover of both PER 1/2 proteins (Akashi et al., 2002; Virshup et al., 2007). Further investigation showed that CKIε-mediated phosphorylation of PER1 at the serine cluster from amino acids 902 to 916 was responsible for masking its NLS motive and subsequently translocation from the nucleus to the cytoplasm of this specific circadian factor (Vielhaber et al., 2000). PER1 degradation is also driven by interaction with β -TRCP1 and β -TRCP2, mediated by CKI/ CK1γ2–(Shirogane et al., 2005; Hirota et al., 2010) (Figure 3, ZT16-24).

CRY proteins complete the repressive complex formed by PERs and CKI, which regulates the negative loop of the circadian clock. Investigations demonstrated that FBXL3 drives CRY ubiquitination and proteasomal degradation (Busino et al., 2007; Godinho et al., 2007; Siepka et al., 2007).

Phosphorylation of CRY1 at serine-588 in the C-term regions seems to stabilize the protein in the nucleus (Figure 3, ZT12-16). Additional studies showed that this phosphorylation diminishes nuclear protein localization and destabilizes the interaction with FBXL3. Although the kinase responsible for Ser-588 phosphorylation is still unknown, it appeared that this modification is negatively regulated by the DNA-dependent protein kinase (DNA-PKcs) (Gao et al., 2013), which promotes CRY1 degradation (Figure 3, ZT16-24).

Moreover, CRY1 can be phosphorylated at the Serine-247 by Mitogen-Activated Protein Kinase (MAPK Kinase). In this specific case, the mutation S-G did not affect CRY1, whereas the mutation S-D impaired the function of inhibiting CLOCK: BMAL1 transcriptional activity. Since this phosphorylation is placed near FAD-contacting amino acid residues, these results would suggest that the amino acid charge of this site is more critical than the phosphorylation *per se* in the regulation of CRY1 activity (Sanada et al., 2004).

Additionally, the same MAPK Kinase can phosphorylate CRY2 at Serine-265 (Ser-265) with the same effect on the regulation of the catalytic activity (Sanada et al., 2004).

On the other hand, the FBXW7-containing SCF complex ubiquitination drives CRY2 degradation. This mechanism requires phosphorylation Thr-300 within the phosphodegron 300-TPPLS-304 (Fang et al., 2015). A schematic summary of the main phosphorylations discussed is shown in Table 1.

**Table 1:** Light-dark related phosphorylation of clock components.

**Protein**	**a.a**.	**Function**	**Kinase**	**Validation method**	**References**
CLOCK	Thr451 Thr461	Promoting nuclear localization	CDK5	- *In vitro* kinase assay - Site-directed mutagenesis	Kwak et al., 2013
	Ser440 Ser441 Ser446	Transactivation	Unknown	- Mass Spectrometry	Robles et al., 2017
	Ser38 Ser42	Inhibition of transactivation	Unknown	- *In vivo* proteomics analysis	Yoshitane et al., 2009
	Ser431	- Primes GSK3 β - Phosphorylation of CLOCK	Unknown	- Site-directed mutagenesis	Spengler et al., 2009
	Ser427	- Nucleus/cytosol shuttling - Degradation	GSK3 β	- Site-directed mutagenesis	Spengler et al., 2009
BMAL1	Ser90	- Heterodimerization with CLOCK - Transactivation	CK2	- *In vitro* kinase assay - *In vivo* phosphospecific antibody	Tamaru et al., 2009
	Ser17 Ser21	Protein degradation	GSK3 β	- *In vitro* kinase assay - Site-direct mutagenesis	Sahar et al., 2010
CRY1	Ser588	Protein stabilization	Unknown	- Site-direct mutagenesis	Gao et al., 2013
	Ser247	Inhibition of CLOCK: BMAL transcriptional activity	MAPK	- *In vitro* kinase assay - Site-direct mutagenesis	Sanada et al., 2004
CRY2	Ser265	Protein stabilization	MAPK	- *In vitro* kinase assay - Site-direct mutagenesis	Sanada et al., 2004
	Thr300	Protein degradation	Unknown	- Indirect	Fang et al., 2015
PER1	- Ser902 - Ser916	Protein degradation	CKI	- *In vitro* kinase assay - *In vivo* mobility shift	Vielhaber et al., 2000
PER2	Ser394	- Heterodimerization with CRY - Nuclear localization - Protein stabilization	CDK5	- *In vitro* kinase assay - Site-direct mutagenesis - Mass Spectrometry - *In vivo* phosphospecific antibody	Brenna et al., 2019
	Ser477	Protein degradation	CKI	- *In vitro* kinase assay	Eide et al., 2005
	Ser-53	Protein degradation	CK2	- *In vitro* kinase assay - Site-direct mutagenesis	Tsuchiya et al., 2009
	Ser659 Ser662 Ser665 Ser671	- Protein stabilization - Phosphoswitch - Temperature compensation	CKI	- *In vitro* kinase assay - Site-direct mutagenesis	Eng et al., 2017

*The color refers to the protein for easier readability*.

## CKI δ/ε -Dependent Phosphoswitch of PER2

Drosophila *double time* (DBT) was the first kinase observed to have a dramatic impact on circadian rhythmicity (Price et al., 1998). DBT shares 86% of identity with the mammalian CKI epsilon, which appears to play a similar role. In 1988, the *Tau* mutation was discovered in the *Syrian hamster*. Later this mutation was identified to be a single nucleotide substitution in the sequence of the *Ck1*ε gene, which changed the protein structure leading to a gain-of-function (Lowrey et al., 2000). These mutant rodents displayed a significant shortening of the circadian period, about 22 h, which resembled the *Per1-2* phenotype (Lowrey et al., 2000; Gallego et al., 2006). Thus, Ck1ε^tau^ was the first mammalian circadian mutant discovered. On the other hand, the *Ck1*ε *null* mutant slightly lengthened the period (Vielhaber et al., 2000).

Since ε and δ are the main CK1 isoforms involved in the circadian regulation, the role of CKI δ in the circadian clock was also studied. *CKI* δ^−/−^ SCN explants (mice showed prenatal lethality) were tested using real-time bioluminescence recording to quantify the circadian period at the cellular level. The results showed a more extended period in *CKI* δ^−/−^ explants compared to both WT and *CKI*ε null mutants. These results suggested a hierarchical organization where CKIδ might be dominant over Ck1ε (Etchegaray et al., 2009).

**Figure 4:**
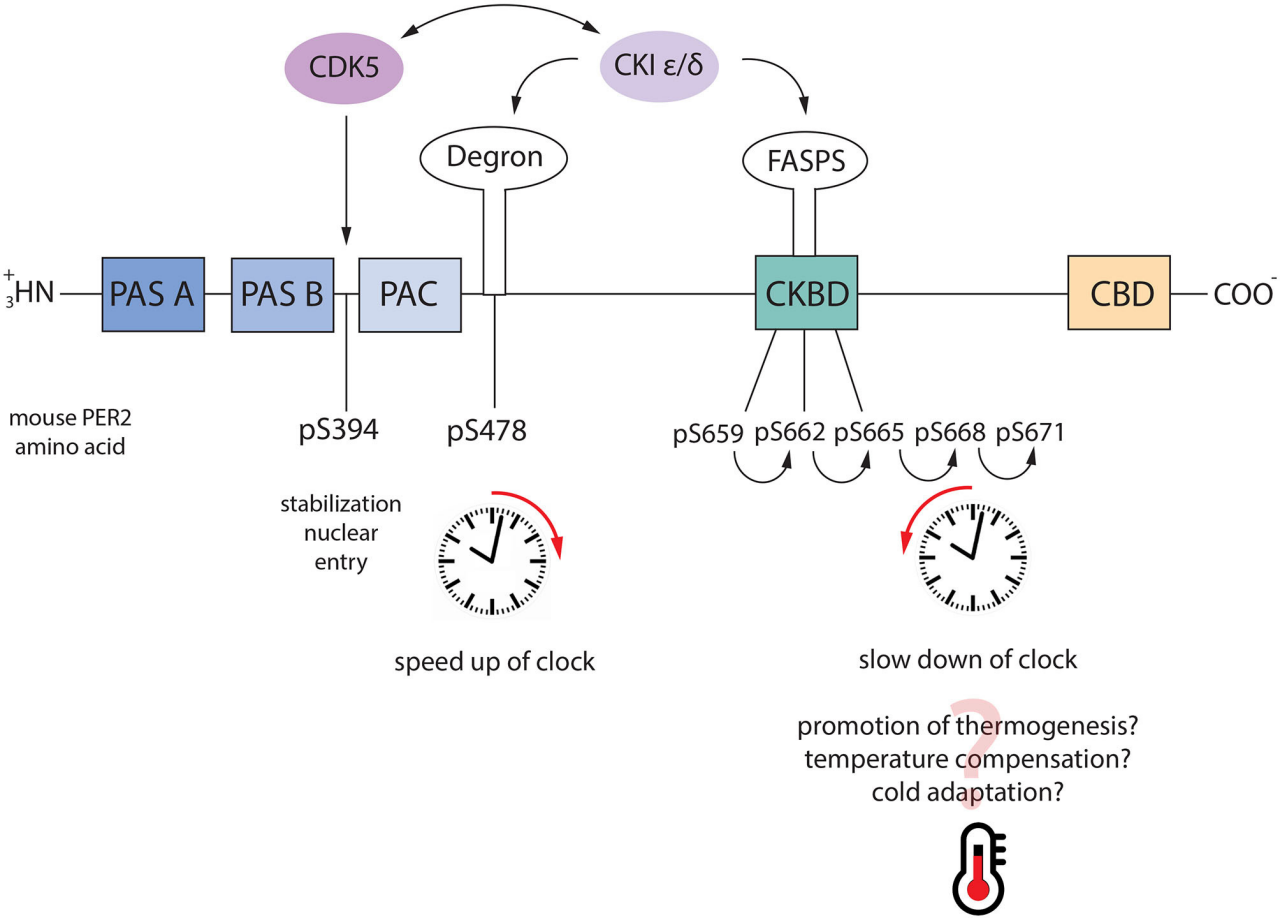
Phosphoswitch model. PER2 regulates the speed of the circadian clock through the axis CDK5-CKI. When PER2 is phosphorylated by CDK5 it is stabilized and it goes into the nucleus. Under normal conditions, nuclear PER2 is phosphorylated by CKI ε/δ at Ser-477 which is followed by nucleus/cytoplasm shuttling and proteasomal degradation. However, following a switch on the temperature, PER2 can be phosphorylated at the FASP sites (pS659-662-665-668-671), which stabilizes the protein. As consequence, the clock is slowed down. Additionally, CDK5 and CKI can reciprocally regulate their activity, speeding up or slowing down the clock accordingly.

CKI can regulate PER2 stability in different ways. CKI-mediated phosphorylation of Serine-477 (Ser-477) was discussed before (see section The Negative Loop). Additionally, due to the -not clear- redundant activity of CK1ε and δ (Lee et al., 2009), both kinases can phosphorylate PER proteins at different sites. For instance, PER2, which contains more than 20 phosphorylation sites (Vanselow et al., 2006), can be sequentially phosphorylated at Serine-659/662/665/668/671 (Shanware et al., 2011) (Figure 4, Table 1). The progressive phosphorylation is dependent on the Ser-659. This specific serine is the priming site, which is phosphorylated by CKIδ/ ε (Narasimamurthy et al., 2018). As previously shown, CKIδ has two splicing variants, CK1δ1 and CK1δ2 (Fustin et al., 2018). CK1δ2, which is 16 aa shorter in the C-terminal region, resembles more CK1ε. Further experiments showed that CK1δ2 and CK1ε are both able to phosphorylate PER2 at Ser-659 better than CK1δ1, *in vitro*, and in cells. These results suggest that the process is tightly connected to the length of the CKI protein's carboxyl terminus (Narasimamurthy et al., 2018). The progressive phosphorylation in this region, called FASP, which is named after the familial advanced sleep phase syndrome (see section Phosphorylation Aberration and Diseases), leads to the stabilization of the PER2 protein. Mutations in the phosphodegron site (Ser-477) led to an extended circadian period in cell culture, while mutation on the priming site at Ser-659 led to faster degradation of PER2 and shorter period length (Vanselow et al., 2006; Masuda et al., 2020). These observations suggest that there is a competition between the two sites (phosphodegron and FASP) for the CKI-mediated phosphorylation. Additionally, the tau mutation—CK1ε gain of function-shows a lower affinity for the FASP region compared to the phopshodegron (Philpott et al., 2020). This observation may explain why PER2 is least stable in mice CK1ε ^tau^ (Meng et al., 2008). However, it is still not completely clear how CKI can choose between the degradation and the stabilization of PER2. The so-called “phosphoswitch model” has been proposed to explain this mechanism, which is based on the original property of CKI kinases that phosphorylate PER2 in a temperature-insensitive manner (Isojima et al., 2009; Zhou et al., 2015; Shinohara et al., 2017).

According to this model, PER2 phosphorylation at Ser-477 is involved in the regular circadian regulation at 30°C. In contrast, the cascade primed by Ser-659 is involved in temperature compensation when the temperature is raised at 37°C. These results would suggest two directions for the phosphoswitch, one toward FASP at a higher temperature, and one toward the phosphodegron at a lower temperature, as also suggested by others (Hirano et al., 2016). However, this conclusion is in contrast with other observations. For instance, PER2 is essential for adaptation to cold temperatures. Mice lacking PER2 were more sensitive to the cold because their thermogenesis system failed to adapt to the new temperature, which means PER2 needs to be stabilized at low temperatures as much as at higher (Chappuis et al., 2013). Thus, another appealing hypothesis might be that once in the nucleus, PER2 can be phosphorylated by CKI through the phosphodegron at serine 477, which drives the protein degradation under constant temperature conditions. When a drastic temperature change stresses the organism, PER2 is stabilized through the phosphorylation cascade within the FASP region to promote more efficient thermogenesis and adaptation (Figure 4).

## Feeding-Driven Phosphorylation of the Clock

The light: dark alternation is the primary circadian entrainment by imposing the timing of sleep/wake cycles. These cycles adjust our behavioral schedules, including feeding time. However, feeding time is the most potent zeitgeber in peripheral clocks. Time-restricted feeding (between ZT5-11 at daytime) can re-entrain circadian liver gene expression in arrhythmic SCN-lesioned mice (Hara et al., 2001). Food can entrain the rhythmicity inducing metabolites production and hormones, whose secretion is controlled by fasting-feeding cycles. For instance, the insulin level is very low during the day, when mice are sleeping (and fasting) while insulin levels increase at the beginning of the night after eating in the active phase. In this paragraph, we discuss the phosphorylation of clock proteins that are connected to the feeding driven oscillations using specific criteria:

- Phosphorylations mediated by kinases that are connected to the mTOR pathway, which is sensitive to the feeding-fasting cycle in the liver (see section Phosphorylation Clock: From the Cellular to the Systemic Level for details);
- Phosphorylation mediated by kinases activated by time-restricted feeding
- Phosphorylation of clock proteins that are proven to be functional only in the liver, but not in the SCN
- Phosphorylation mediated by kinases which affect the food intake and feeding schedule
- Phosphorylation mediated by kinases that are involved in the whole-body lipid/glucose homeostasis.

It has been demonstrated that AKT can be activated upon the increased level of insulin through the PI3K/AKT pathway (see section Phosphorylation Clock: From the Cellular to the Systemic Level), and therefore phosphorylate CLOCK at Serine-845.

**Figure 5:**
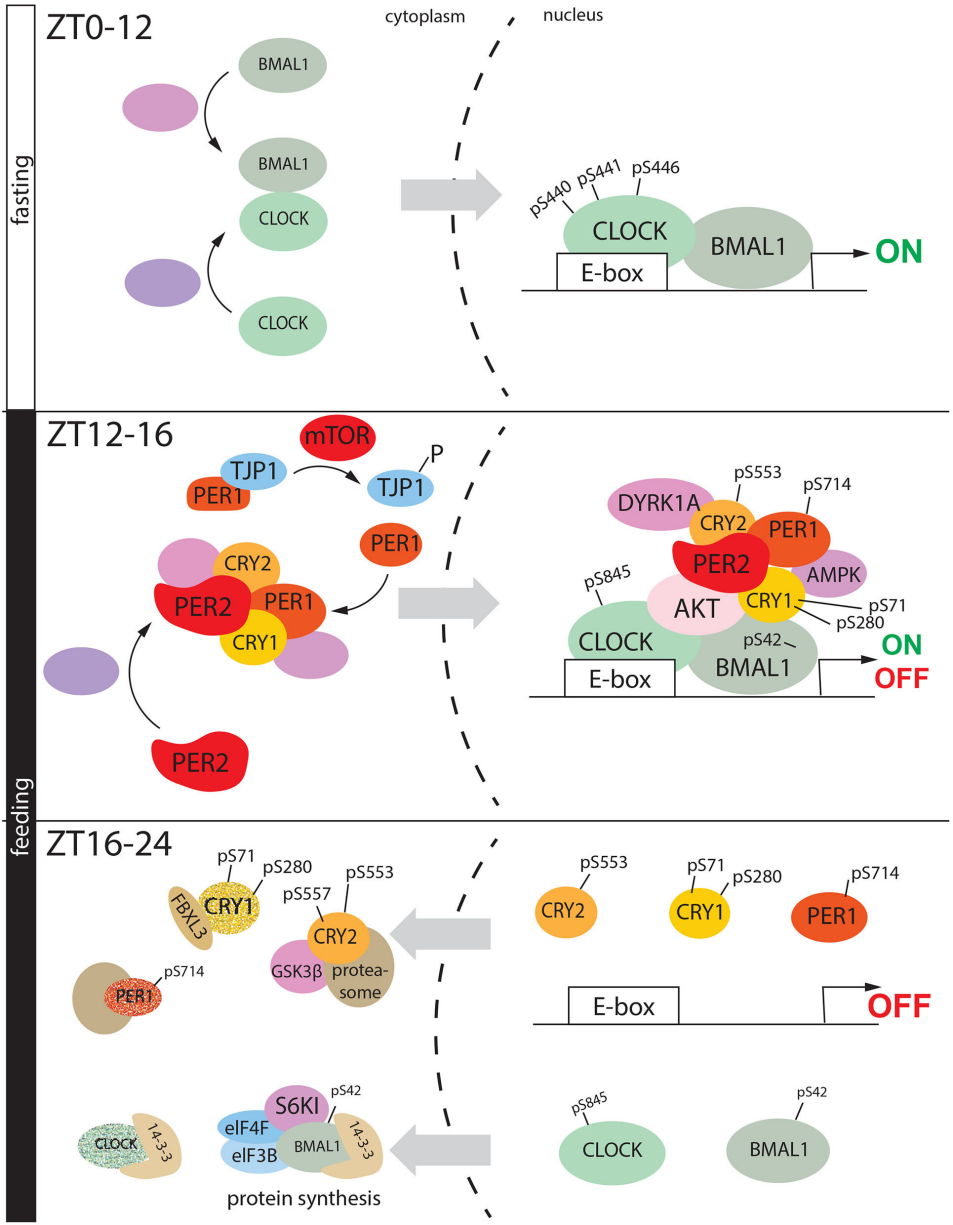
Feeding regulates phosphorylation mediated circadian cycle in liver. During the fasting period, CLOCK and BMAL heterodimer goes into the nucleus, and here upon phosphorylation they initiate the gene expression. During the early night, in the transition between fasting and feeding a nuclear macromolecular complex is assembled on the promoter of clock-controlled genes, formed by CLOCK, BMAL1, PER2, PER1, AKT (activated by insulin) DYRK1A, CRY1, CRY2, and AMPK. As consequence, CKOCK is phosphorylated at serine 845, BMAL1 at serine 42, CRY1 at serine 71 and 280 (AMPK), PER1 at serine 714. All these events drive the transition from transactivation to gene repression. BMAL1 pSer-42 translocated into the cytoplasm where it is phosphorylated again at serine 42 by S6K1. This signal triggers protein synthesis which is driven by the complex formed by BMAL1, S6KI, 14-3-3, eIF4F, and eIF3B. On the other hand, CLOCK (pSer-845), CR2 (pSer-553, pSer-557), CRY (pSer-71, pSer280), PER1 (pSer-714) are degraded in the cytoplasm, via proteasome.

This phosphorylation site was originally identified as an ASK kinase-dependent and cellular stress-responsive phosphorylation site (Imamura et al., 2018). This modification appears to be responsible for the shuttling of the protein from the nucleus to the cytoplasm (Luciano et al., 2018) (Figure 5, ZT12-16). Here, CLOCK binds 14-3-3, which confines the circadian protein in the cytoplasm (Noguchi et al., 2018) (Figure 5, ZT16-20). CLOCK S845A mice showed a regular clock in the SCN but not in the peripheral tissues, suggesting that this specific phosphorylation is more important in the liver than in the SCN (Luciano et al., 2018).

Insulin can modulate the nuclear accumulation of BMAL1 in hepatocytes via phosphorylation by AKT at Serine 42 (Ser-42), promoting its dissociation from the chromatin and cytoplasmic confinement driven by 14-3-3 (Figure 5 ZT12-16) (Dang et al., 2016). Subsequently, BMAL1 is phosphorylated by Ribosomal protein S6 kinase beta-1 (S6K1) again at Ser-42 through the insulin- mammalian target of rapamycin (mTOR) pathway (see section Phosphorylation Clock: From the Cellular to the Systemic Level). BMAL1 rhythmically associates with S6K1 and the translational machinery in the cytoplasm, and the phosphorylation at Ser-42 is required to promote protein translation, closing the transcriptional-translational feedback loop of the circadian clock (Lipton et al., 2015) (Figure 5, ZT16-24).

On the other hand, under restricted feeding conditions (access to food only 4-h during the light phase), Protein Kinase Cγ (PKCγ) can phosphorylate BMAL1. This modification reduces the ubiquitination and stabilizes the protein, causing a circadian phase shift (Zhang et al., 2012). PKCγ seems to be very sensitive to the food intake, since its daily activation profile changes during the restricted feeding compared to *ad libidum*, in mice kept under 12:12 light/dark conditions. PKCγ-mediated phosphorylation of BMAL1 seems dispensable during the normal light: dark entrainment, but it seems to be an essential regulator of BMAL1 under restricted feeding. AKT (which is activated by insulin increase) can modulate GSK3β through the pathway AKT- GSK3β, and this can counteract PKCγ activity on BMAL1. These observations suggest that food can synchronize the circadian clock through the axis GSK3β-BMAL1-PKCγ.

The mTOR pathway regulates PER1 nuclear shuttling in the liver. After nighttime feeding, mTOR phosphorylates Tight Junction Protein 1 (TJP1). This protein is located on a cytoplasmic membrane surface of tight intercellular junctions, where it traps PER1. Once the mTOR pathway is active, the heterodimer TJP1: PER1 is disassembled, promoting PER1 nuclear localization. Here, the clock factor can inhibit CLOCK: BMAL1 transcriptional activity (Liu et al., 2020).

Phosphorylation of PER1 at Serine-714 (Ser-714) in the liver is associated with the regulation of feeding rhythms and food intake (Liu et al., 2014) (Figure 5, ZT12-16). PER1^S714G^ mice exhibit an advanced phase of feeding behavior, a propensity to obesity on a high-fat diet. At the molecular level, it has been proposed that pSer-714 is necessary to keep “on time” the feedback loop, and PER1^S714G^ mice showed an accelerated speed of the cycle.

By mass spectrometry, and subsequently biochemical experiments, it was observed that phosphorylation mediated by 5′-adenosine monophosphate-activated protein kinase (AMPK), in the liver, at Serine-71 (Ser-71) and, to a lesser extent, at Serine-280 (Ser-280) are involved in the regulation of CRY1 stability (Lamia et al., 2009) (Figure 5, ZT12-26). Due to the role of AMPK as a fasting sensor (see section Phosphorylation Clock: From the Cellular to the Systemic Level for details), this evidence proved a direct connection between metabolism and the circadian clock. The diurnal peak of CRY1 protein accumulation is in antiphase with one of AMPK, supporting the idea that nuclear AMPK can drive CRY1 degradation (Lamia et al., 2009) (Figure 5, ZT16-20).

Rescue experiments performed *in vitro* on HEK 293, where *Cry1* was suppressed, showed that CRY1 S to G 71 was not able to restore the circadian rhythmicity compared to wt CRY1 (Liu and Zhang, 2016). However, an extensive investigation performed on mice mutant for Ser-71 led to evidence that the specific phosphorylation site does not show any impact on *in vivo* regulation of the circadian behavior (Vaughan et al., 2019). These observations raise the question of whether CRY1 phosphorylation works only as a metabolic sensor.

Hypothalamic Dual specificity tyrosine-phosphorylation-regulated kinase 1a (Dyrk1a) regulates food intake in mice (Hong et al., 2012). CRY2 is phosphorylated by DYRK at Ser-557 in the mouse liver, and this modification rhythmically oscillates over the day (Kurabayashi et al., 2010). Then, pSer557-CRY2 is used as a priming site for GSK3 beta-mediated phosphorylated at Ser-553 (Harada et al., 2005), suggesting that the axis DYRK-GSK3 beta can affect CRY2 stability (Kurabayashi et al., 2010). A point mutation at Ser-557 stabilizes the protein, thus lengthening the period (Hirano et al., 2014). Additionally, pSer-557 CRY2 colocalized mostly into the nucleus, suggesting that the specific phosphorylation drives CRY2 degradation by multiple steps. In detail, GSK-3β-mediated CRY2 phosphorylation is primed by DYRK1A, which phosphorylates the protein at the amino acid Ser-557 (Kurabayashi et al., 2010) (Figure 5 ZT12-16). This phosphorylated form can bind GSK-3β, which drives the degradation pathway of CRY2 via Ser-553 phosphorylation (Figure 5, 16-24). However, the E3 ligase involved in these pathways is still unknown. Due to the role of DYRK1A in the regulation of food intake, the phosphorylation of CRY2 might suggest a further connection between circadian rhythms and feeding.

REV-ERBα is a nuclear receptor involved in the stabilizing loop of the circadian clock mechanism with a role as s transcriptional repressor. It exerts its functions through binding to genomic response elements (termed *ROREs*). This kind of regulation is balanced by the positively acting orphan nuclear receptors RORa (Sato et al., 2004; Takeda et al., 2012).

REV-ERBα is also phosphorylated at Threonine-275 (Thr-275) by Cyclin-Dependent-Kinase 1 (CDK1) in the mouse liver (Zhao et al., 2016). CDK1-mediated phosphorylation drives the degradation of REV-ERBα via F-Box and WD Repeat Domain Containing 7 (FBXW7). The phosphorylation at Thr-275 oscillates during the day, with a peak during the early morning, when the total protein amount is lower. Knock out of both CDK1 and FBXW7 impairs the circadian rhythmicity's amplitude. As a consequence, expression of genes involved in lipid and glucose metabolism, such as Insig2 and G6Pase, is aberrant, influencing liver steatosis, and gluconeogenesis. These results altogether showed the importance of post-translational regulation as coordinator of the body energy and homeostasis.

Finally, CDK9 interacts with REV-ERBα and attenuates the binding to the *RORE* elements.

A schematic summary of the main phosphorylations discussed is shown in Table 2.

**Table 2:** Feeding-related phosphorylation of clock components.

**Protein**	**a.a**.	**Function**	**Kinase**	**Validation method**	**References**
CLOCK	Ser845	- Nucleus/cytosol shuttling - Dimerization with 14-3-3	AKT	- *In vitro* kinase assay - Site-direct mutagenesis	Luciano et al., 2018
BMAL1	Ser42	Nucleus/cytosol translocation	AKT	- *In vitro* kinase assay - Site-direct mutagenesis - Mass Spectrometry	Dang et al., 2016
		Initiation of ribosomal translation	S6K1	- *In vitro* kinase assay - Site-direct mutagenesis - Mass Spectrometry - *In vivo* phosphospecific antibody	Lipton et al., 2015
CRY1	Ser71 Ser280	Protein degradation	AMPK	- *In vitro* kinase assay - Site-direct mutagenesis - Mass Spectrometry	Lamia et al., 2009
CRY2	Ser553	Protein degradation	GSK3 β	- *In vitro* kinase assay - Site-direct mutagenesis	Kurabayashi et al., 2010
	Ser557	Protein degradation	Dyrk1a	- *In vitro* kinase assay - Site-direct mutagenesis	Kurabayashi et al., 2010
PER1	Ser714	Protein degradation	Unknown	- Indirect	Liu et al., 2014
Rev-Erbα	Thr275	Protein degradation	CDK1	- *In vitro* kinase assay - Site-direct Mutagenesis - *In vivo* phosphospecific antibody	Zhao et al., 2016

*The color refers to the protein for easier readability*.

## Phosphorylation Clock: From the Cellular to the Systemic Level

In the last few years, large-scale analysis of post-translational modifications, such as phosphorylation, became accessible to most of the laboratories. The first circadian phosphoproteome database was obtained from the liver, which is the tissue that displays the most robust circadian activity, together with the SCN (Robles et al., 2017). Interestingly, 25% of the phosphopeptides on over 40% of the identified phosphoproteins showed oscillations over 24 h. This large amount of oscillating phosphopeptides was highly astonishing, considering that only 10% of the total transcriptome displayed circadian oscillation in gene expression (Panda et al., 2002a; Storch et al., 2002). The amplitude of the circadian phosphoproteome was four times higher than the one of the entire liver proteome (Robles et al., 2014, 2017). These results suggested that phosphorylation is one of the most robust markers of rhythmicity in this organ.

Interestingly, the oscillating phospho-sites that were identified from the Mass-Spec belonged to signaling pathways involved in insulin signaling, autophagy, circadian rhythms, and TGF-beta signaling. These findings supported the hypothesis that post-translational modification connects circadian rhythms with physiological processes. Phosphopeptide oscillations showed peaks in two different temporal windows around the day, one around the circadian time (CT) 4–6 when the light is on, and the second around CT 15–17 when the light is off. These peaks were in opposition to the ones observed for the total proteome (Robles et al., 2014, 2017). These pieces of evidence suggested that there is a group of kinases activated during the resting/fasting phase (corresponding to the light on) and another activated during the active/feeding period (corresponding to the light off).

An example of a phosphorylation cascade, that is activated during the light phase is the AMPK signaling pathway. The adenosine monophosphate (AMP)–activated protein kinase (AMPK) is a serine/threonine kinase, which is activated by ATP exhaustion that leads to an increase of AMP, a usual signal of cellular stress (Hardie, 2007). The AMPK cascade is under the control of both circadian clock and feeding, which couples metabolic state with circadian rhythmicity (Suter and Schibler, 2009). During the sleeping phase [zeitgeber time (ZT) 0–12, light on], the AMP/ATP ratio continuously increases up to ZT12. The AMP accumulation increases in the cells and the factor binds AMPK. This binding provokes a change of conformation in the AMPK quaternary structure.

**Figure 6:**
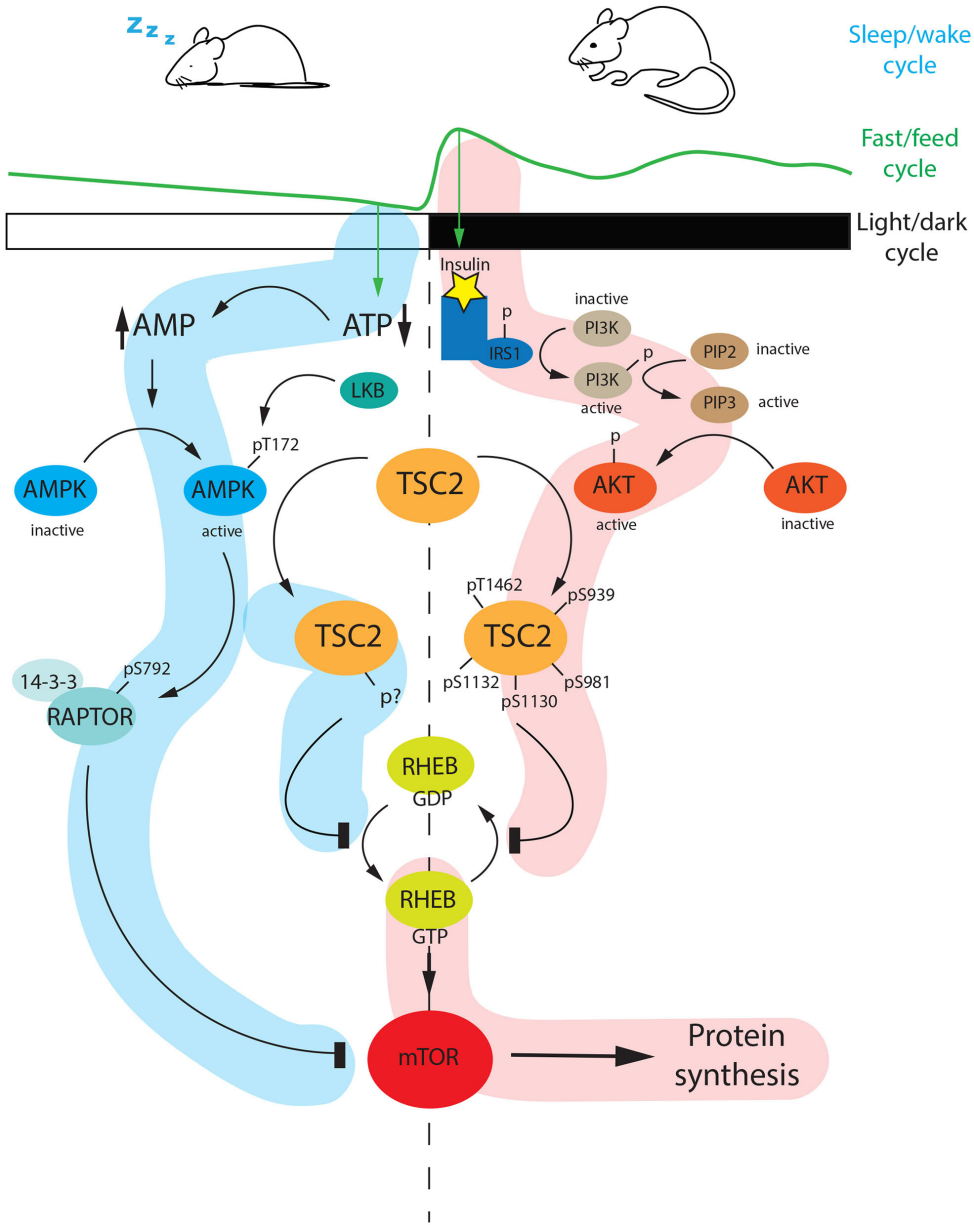
Diurnal regulation of the mTOR pathway. During the light phase (ZT0-ZT12), mice are sleeping and therefore fasting. Fasting is a specific stimulus that drivers AMP accumulation at the expense of ATP. AMP accumulation triggers AMPK activation via phosphorylation at Threonine 172 (Thr-172) mediated by LKB1. The active AMPK can phosphorylate RAPTOR at serine 792 (Ser-792) which subsequently interacts with 14-3-3 and inhibits mTOR. In parallel, AMPK can phosphorylate TSC2 at an unknown phosphosite. The phosphorylated TSC2 inhibits the conversion of RHEB-GDP (inactive) to the active form RHEB-GTP, which is a positive regulator of mTOR. As a consequence, the mTOR pathway is shut down. On the other hand, at night (ZT12-ZT24) when mice are active and they are feeding, the internal level of insulin is increasing. This event promotes the PI3K-AKT cascade, which ends with an active form of AKT phosphorylating TSC2 at different sites (Thr-1462, Ser-939, Ser-981, Ser-1130, Ser-1132). The hyperphosphorylated TSC2 inhibits the conversion of RHEB-GTP to RHEB GDP, thus the active RHEB-GTP can turn on the mTOR pathway. AMP, Adenosine MonoPhosphate; ATP, Adenosine TriPhosphate; LKB1, Liver Kinase B1; AMPK, 5′ Adenosine Monophosphate-activated Protein Kinase; TSC2, Tuberous Sclerosis Complex 2; RHEB, Ras Homolog Enriched in Brain; GTP, Guanosine TriPhosphate; GDP, Guanosine DiPhosphate; IRS1, Insulin Receptor Substrate 1; PI3K, PhosphatidylInositol-3-Kinase; PIP2, Phosphatidylinositol 4,5-bisphosphate; PIP3, Phosphatidylinositol (3,4,5)-trisphosphate; AKT, AKR Thymoma; mTOR, mammalian Target Of Rapamycin; p, phosphorylated; s, serine.

As a consequence, the Threonine-172 (T-172) is exposed, and it can be phosphorylated by Liver Kinase B1 (LKB1), which itself is sensitive to changes in AMP/ATP levels. This specific modification activates AMPK, which subsequently phosphorylates many targets. This mechanism is a direct readout of diurnal metabolic changes (Shackelford and Shaw, 2009; Lee and Kim, 2013) (Figure 6, light phase).

For instance, upon phosphorylation, AMPK positively regulates the NAMPT accumulation. NAMPT is one of the essential molecular connectors between metabolic changes and circadian regulation in peripheral tissues (Nakahata et al., 2009; Ramsey et al., 2009). This accumulation enhances the Silent Information Regulator 1 (SIRT1) histone deacetylase activity, which then regulates the molecular clock (Asher et al., 2008; Nakahata et al., 2008).

Another example of how phosphorylation ties metabolism to circadian rhythm is the AMPK-mTOR-AKT axis (Figure 6). The mammalian target of rapamycin (mTOR) pathway is a crucial point in the regulation of metabolism and physiology in mammals (Laplante and Sabatini, 2012). mTOR presents at least two different multiprotein complexes, mTORC1 and mTORC2. The Regulatory-associated-protein of mTOR (RAPTOR) is a member of the mTOR cascade signaling. In the absence of nutrients (morning fasting), AMPK is active, and it phosphorylates RAPTOR at serine-792 (Ser-792). The phosphorylated protein can subsequently bind 14-3-3, which belongs to the family of regulatory molecules, and this increases the affinity of RAPTOR for mTORC1 (Xu et al., 2012).

As a consequence, this cascade leads to the inhibition of mTORC1 during the light phase. Additionally, AMPK can phosphorylate Tuberous Sclerosis Complex 2 (TSC2). This form of TSC2 can inhibit the conversion of Ras homolog enriched in the brain (RHEB, a GTP-binding protein) from GDP to GTP-bound. The RHEB-GDP is not able to activate the mTOR pathway.

On the other hand, the mTOR pathway is positively regulated at night by AKT. AKT, also known as PKB, is a cytosolic kinase involved in the Phosphoinositide 3-kinase (PI3K)-AKT pathway. In the presence of specific ligands, for instance, an increased level of insulin, phosphoinositide 3-kinase (PI3K) is activated. The PI3K cascade promotes AKT phosphorylation, and therefore activation. Active AKT phosphorylates TSC2 at different sites (Ser-939, Ser-981, Thr-1462, Ser-1130, Ser-1132). This modified form of TSC2 inhibits the conversion of RHEB-GTP to RHEB-GDP. Thus RHEB in its GTP-bound form can trigger the mTOR pathway to promote ribosome biogenesis, which couples circadian phosphorylation and energy stress (Jouffe et al., 2013; Robles et al., 2017; Cao, 2018). Altogether, these observations show that the AMPK-AKT axis regulates the mTOR pathway in a circadian way mediating the transition from the resting phase to the active phase in mice.

## Phosphorylation Aberration and Diseases

Phosphorylation is an essential mechanism for modulating biological responses. Alteration of kinases activity or point mutations on target sequence can lead to many diseases as, for instance, Advanced Sleep Phase Disorder (ASPD) (Auger, 2009). The Familial Advanced Sleep Phase Disorder (FASP) is the most famous case belonging to the ASPD. Subjects with this disease show an alteration of the circadian period of free-running, which is shorter than healthy individuals.

As a consequence, it causes an earlier onset and offset, which impacts the social life of these individuals. The first clock-gene mutation causing FASP discovered was the hPER2 S662G (mPER2 S659), which destabilizes the protein causing a shortening of the period length (Toh et al., 2001).

Additionally, PER1 S714G also is involved in the FASP showing advanced sleep-wake rhythms (Liu et al., 2014). Both human PER1 and PER2 are phosphorylated, and they interact with CK1ε (Keesler et al., 2000; Camacho et al., 2001). As also expected, CKIδ mutation (Thr-44A) causes FASP (Xu et al., 2005). On the other hand, a mutation of CRY1 is responsible for Delayed Sleep Phase Disorder (DSPD). This mutation causes a deletion of 24 amino acids within the C-Term region, which is an essential target of kinases. As a consequence, CRY is more abundant in the nuclei, causing a lengthening of the period (Patke et al., 2017).

CDK5 is an essential kinase involved in neurogenesis. Aberration in CDK5 kinase activity leads to Neuro Degenerative diseases (NDs) (Kawauchi, 2014). Since CDK5 kinase activity shows a diurnal profile (peaking during the night phase), we can assume that alteration in the day-night cycles can be responsible for ND malignancies. CKI was discovered to be aberrant in NDs, suggesting a further connection between circadian kinase activity and diseases (Schwab et al., 2000). Alteration of GSK-3β is involved in mood disorders, which have been strongly connected to circadian disorders (Li and Jope, 2010).

Many forms of cancer are connected to circadian aberrations. For instance, alteration in the pathway of CRY1 Thr-300 phosphorylation within the phosphodegron is associated with the chemoresistance of colorectal cancer (Fang et al., 2015). The mTOR pathway alteration is involved in many forms of cancer as well, which are connected to the circadian clock at the epidemiological level. Since obesity or generally, metabolic disorders are often associated with cancer development, the role of the mTOR pathway, which is regulated by the daily oscillation between fasting and feeding, might connect these different health issues (Swierczynska and Hall, 2016). However, many therapies nowadays imply the use of drugs that can target these kinases modifying even the circadian clock. For instance, the photocaged longdaysin is a purine-based inhibitor of CKIα, CKIδ and ERK2 that increases circadian period in cells (Hirota et al., 2010; Kolarski et al., 2019). Additionally, the compound PF670462 inhibits Casein Kinase 1 δ/ε and lenghtens circadian period (Meng et al., 2010). Furthermore, this inhibitor has anti-fibrogenic effects (Keenan et al., 2018). Nevertheless, the question remains whether these therapies should require a chrono-pharmacological approach due to the difference between subjects in terms of daily expression of proteins that are the target of these drugs (Griffett and Burris, 2013).

## Conclusions

Here we described the role of phosphorylation on the circadian clock and vice versa. Although the transcriptional-translational feedback loop is considered as a central dogma for the circadian clock regulation (Hurley et al., 2016), many pieces of evidence suggest that it is not the most critical mechanism for coordinating rhythmicity. It has been demonstrated that the rhythmicity of mRNA is not necessarily predictive of the rhythmicity of its cognate protein, and proteins with a non-cyclic accumulation over the day can still generate rhythmic responses through PTM modifications (Mauvoisin et al., 2014). For instance, post-translational rhythms were observed in enucleated human red blood cells without oscillating mRNA (O'Neill and Reddy, 2011). This is a quite conserved mechanism across species. For instance, it has been shown that the phosphorylation of FRQ, which is the negative regulator of the circadian feedback loop, rather than its quantity, is crucial for the determination of the circadian period in *Neurospora* (Larrondo et al., 2015). Altogether these observations highlight the relevance of PTM modifications, such as phosphorylation, in the generation of circadian rhythmicity, which seems to be as crucial as the transcriptional-translational paradigm. Additionally, we emphasized the role of the phosphorylation in metabolic pathways tied to the molecular clock and how dangerous for health the disruption of such mechanisms can be. Many compounds can regulate the activity of kinases. For instance, specific drugs can block the PI3K signaling (described in this review) inhibiting tumor growth (Jamieson et al., 2011). However, because this specific pathway (not only this) is driven by insulin peak associated with the circadian food intake, it would also be probably necessary to dose the drug at the appropriate time during the day, to be more successful. This aspect, time-related and not only dose-related, belongs to the growing field of the chrono-pharmacology, called “the medicine in the fourth dimension,” which is the future of the therapeutic approaches to diseases (Cederroth et al., 2019).

## Data Availability Statement

The original contributions presented in the study are included in the article/supplementary material, further inquiries can be directed to the corresponding author.

## Author Contributions

AB and UA conceived and wrote the manuscript. Both authors contributed to the article and approved the submitted version.

## Conflict of Interest

The authors declare that the research was conducted in the absence of any commercial or financial relationships that could be construed as a potential conflict of interest.

## Abbreviations

PTM: Post-Translational Modifications
CDK: Cyclin Dependent Kinase
GSK3β: Glycogen Synthase Kinase 3 beta
CK2α: Casein Kinase 2 Alpha
CK2β: Casein Kinase 2 Beta
UBE3A: Ubiquitin Protein Ligase E3A
PFK: Periodic Fluctuant Kinase
SCF: Skp-Cullin-F bofbx
CKI δ/ε: Casein Kinase I Delta/Epsilon
β-TrCP: Beta-transducin repeats-containing proteins
FBXL3: F-Box And Leucine Rich Repeat Protein 3
DNA-PKcs: DNA-dependent protein kinase
MAPK Kinase: Mitogen-Activated Protein Kinase
DBT: Drosophila *double time*
FASP: familial advanced sleep phase syndrome
S6K1: Ribosomal protein S6 kinase beta-1
mTOR: Mammalian target of Rapamicin
PKCγ: Protein Kinase Cγ
TJP1: Thight Junction Protein 1
AMPK: 5′-adenosine monophosphate-activated protein kinase
DYRK1A: Dual specificity tyrosine-phosphorylation-regulated kinase 1A
FBXW7: F-Box And WD Repeat Domain Containing 7
TGF: Transforming Growth Factor
LKB1: Liver Kinase B1
AMP: Adenosine MonoPhosphate
ATP: Adenosine Triphosphate
NAMPT: Nicotinamide Phosphoribosyltransferase
Sirt1: Silent Information Regulator 1
Regulatory-associated-protein of mTOR: RAPTOR
TSC2: Tuberous Sclerosis Complex 2
RHEB: Ras homolog enriched in the brain
PI3K: Phosphoinositide 3-kinase
GTP: Guanosine-5′-triphosphate
GDP: Guanosine diphosphate
ASPD: Advanced Sleep Phase Disorder
FASP: Familial Advanced Sleep Phase Disorder
DSPD: Delayed Sleep Phase Disorder.
